# Pulmonary Veno-Occlusive Disease: A Comprehensive Review of Diagnostic Challenges, Therapeutic Limitations, and Evolving Management

**DOI:** 10.3390/arm93060048

**Published:** 2025-10-31

**Authors:** Brian Foster, Sikandar Khan, Ana Suarez Gonzalez, Samantha Gillenwater

**Affiliations:** Department of Pulmonary Disease and Critical Care Medicine, Cleveland Clinic Florida, Weston, FL 33331, USA; khans31@ccf.org (S.K.); suareza9@ccf.org (A.S.G.); gillens@ccf.org (S.G.)

**Keywords:** pulmonary veno-occlusive disease, PVOD, pulmonary arterial hypertension, PAH, pulmonary vascular remodeling, *EIF2AK* mutation, lung transplantation

## Abstract

Pulmonary veno-occlusive disease (PVOD) is a rare and under-recognized cause of pulmonary hypertension. It is characterized by fibrotic obstruction of small pulmonary veins and venules. Its clinical presentation closely mimics pulmonary arterial hypertension (PAH), leading to frequent misdiagnosis, delayed recognition, and potentially harmful exposure to PAH-specific vasodilator therapy. This review aims to synthesize our evolving understanding of PVOD, discussing its etiologies, role of genetic underpinnings, histopathologic features, pathophysiology, clinical presentation, and characteristic imaging findings. It then discusses management strategies emphasizing early recognition, supportive care, avoidance of inappropriate PAH therapies due to poor response, and timely referral for lung transplantation. Despite advances in identification and management, PVOD remains a fatal condition with a median survival of less than two years, underscoring the importance of early recognition and multidisciplinary care.

## 1. Introduction

Pulmonary veno-occlusive disease (PVOD) is a rare and often misdiagnosed cause of pulmonary hypertension, marked by progressive obstruction of the pulmonary venous and capillary circulation. This leads to increased pulmonary vascular resistance, right heart failure, and ultimately, death. First described in 1934 at the University of Munich by Dr. Julius Höra, PVOD was recognized as a distinct entity in 1966, differentiating it from other forms of pulmonary hypertension, particularly idiopathic pulmonary arterial hypertension (IPAH) [1,2].

Nearly a century later, PVOD continues to challenge clinicians and researchers alike. Its subtle clinical presentation and paradoxical response to standard PAH therapies make it a uniquely difficult condition to identify and manage. Although advances in imaging, genetics, and transplant medicine have improved recognition and treatment, many fundamental questions remain unanswered. This review explores the current understanding of PVOD, from pathophysiology and diagnostic criteria to treatment strategies and future directions.

## 2. Epidemiology and Clinical Importance

PVOD is an exceptionally rare cause of pulmonary hypertension with an estimated annual incidence rate that is hard to approximate. Conservative estimates based on prior histological series of patients with hypertensive pulmonary vascular disease suggest that PVOD accounts for 3–12% of cases labeled as “idiopathic PAH” ante mortem [3,4,5]. More recent data suggests this number is closer to 10% and propose an annual incidence rate of 0.1 to 0.2 cases per million persons in the general population [5,6]. PVOD affects all age groups, although it most commonly affects individuals between the second and fourth decades of life. Although the sporadic form predominantly affects older age males [2], the heritable form has been shown to approach a 1:1 ratio of affected men to women, different from classical primary pulmonary hypertension and IPAH [6].

The clinical importance of PVOD lies not only in its progressive and fatal course but in its potential for rapid decompensation, particularly in response to inappropriate PAH therapies. This critical distinction underscores the necessity for early and accurate diagnosis to guide appropriate management and improve patient outcomes as the natural history is marked by worsening dyspnea, hypoxemia, and right heart failure, with a median survival of 12–24 months following diagnosis in the absence of lung transplantation [7].

Given its low prevalence, nonspecific presentation, and risk of clinical worsening with standard PAH therapies, PVOD requires a high index of suspicion and early referral to specialized centers for accurate diagnosis and management.

## 3. Pathophysiology

The pathogenesis of PVOD centers on progressive fibrotic narrowing and occlusion of small pulmonary veins and venules, resulting in post-capillary vascular obstruction [6]. This leads to elevated pulmonary capillary hydrostatic pressure, interstitial edema, and pulmonary capillary congestion. Histologically, PVOD is characterized by intimal fibrosis of pulmonary venules, often accompanied by remodeling of alveolar capillaries and, to a lesser extent, small pulmonary arteries [8]. Intimal thickening mostly involves venules and small veins within lobular septa. This thickening can be loose and edematous in earlier stages, progressing to dense and sclerotic fibrosis, eventually leading to complete venous occlusion as the closely apposed medial layer of affected veins may become arterialized due to increased elastic fibers that over time can become encrusted with calcium [6]. This pattern of venous and capillary involvement distinguishes PVOD from IPAH, which primarily affects precapillary arterioles. Although the primary pathology resides in the venous system, secondary changes can occur in the pulmonary arterioles, including moderate to severe medial hypertrophy in approximately one-half of cases; however, features such as arteritis and plexiform lesions are typically absent in PVOD [9]. The resulting downstream pressure overload predisposes to occult alveolar hemorrhage and explains the risk of pulmonary edema with vasodilator therapy, as increased flow through an obstructed venous circuit exacerbates capillary transudation and fluid accumulation. Over time, these changes contribute to pre-capillary pulmonary hypertension, impaired gas exchange, and progressive right ventricular dysfunction.

## 4. Etiology

While a portion of PVOD cases remain idiopathic, an expanding body of evidence links the disease to a range of pre-disposing factors that can be grouped into genetic, toxic-environmental, infectious, and systemic categories.

### 4.1. Genetic Predisposition

The most significant breakthrough in understanding PVOD etiology has been the discovery of biallelic mutations in the EIF2AK4 gene, now considered diagnostic of heritable PVOD [10]. These mutations follow an autosomal recessive inheritance pattern. EIF2AK4 encodes for eukaryotic translation initiation factor 2 alpha kinase 4 (GCN2), a serine/threonine-protein kinase that is a key sensor in the integrated stress response (IRS) pathway. Mutations in EIF2AK4 are found in a substantial proportion of heritable PVOD cases and are also present in some sporadic forms (albeit rare), highlighting a strong genetic predisposition [10]. Although precise mechanisms are still being determined, biallelic loss-of-function mutations in EIF2AK4 result in the absence or dysfunction of GCN2, thereby disrupting normal endothelial cell stress response, leading to abnormal proliferation, angiogenesis, and vascular remodeling [10,11,12].

Beyond *EIF2AK4*, several other genetic and molecular pathways have been implicated in PVOD pathogenesis, though they are not currently considered diagnostic. Rare variants in PAH-associated genes including *BMPR2*, *TBX4*, and *SMAD9* have been identified in patients with PVOD-like histology [13,14,15,16,17,18], raising the possibility of phenotypic overlap or the presence of disease-modifying alleles. While *BMPR2*, *TBX4*, and *SMAD9* mutations have occasionally been identified in patients with PVOD-like features, they are exceedingly rare, and their clinical relevance remains limited in PVOD, contrasted to the central role of *EIF2AK4* mutations.

### 4.2. Toxic and Environmental Exposures

Occupational and iatrogenic exposures are among the most well-documented contributors to non-heritable PVOD. Two primary exposure categories have been implicated: organic solvents and chemotherapeutic agents. Epidemiologic studies have consistently identified trichloroethylene as the most strongly associated organic solvent, although other halogenated hydrocarbons may also contribute [19]. Chronic exposure is thought to include endothelial injury, initiating a cascade of venular remodeling and fibrosis, ultimately leading to the development of PVOD. In parallel, several alkylating and antitumor chemotherapeutic agents, most notably mitomycin C, have been linked to PVOD development [20,21]. Other agents, including cyclophosphamide, carmustine, and busulfan, have also been implicated in case series. These agents exert cytotoxic effects on the pulmonary endothelium, leading to progressive venous occlusion.

### 4.3. Infectious Agents

While less clearly defined, some infectious agents have been proposed as potential contributors to the development of PVOD. Among these, HIV is the most frequently reported; however, the pathophysiologic link remains speculative [22,23]. It is hypothesized that chronic immune activation, direct viral endothelial injury, and/or co-infection play a role in venular remodeling. Other viral infections have only been anecdotally associated with the development of PVOD though these findings raise the possibility that chronic or latent viral infections may serve as initiating triggers in susceptible individuals, particularly in the setting of immunosuppression or prior vascular injury. Nonetheless, no single pathogen has been definitively linked to PVOD pathogenesis, and further research is needed to clarify the role of infectious agents in disease development.

### 4.4. Systemic and Autoimmune Diseases

PVOD can occur in association with various systemic diseases, suggesting a complex interplay of underlying inflammatory and immune-mediated processes. Connective tissue diseases, such as systemic sclerosis, systemic lupus erythematosus, and mixed connective tissue disease, are notable associations. In particular, a significant proportion of patients with PAH secondary to systemic sclerosis have been found to have radiographic and pathological evidence of venous involvement consistent with PVOD [24]. Other systemic conditions linked to PVOD include sickle cell disease and sarcoidosis [25,26]. Certain malignancies, including Hodgkin’s lymphoma and some primary lung cancers, have also been reported in association with PVOD, although development of PVOD may have resulted from radiotherapy and chemotherapy treatment, rather than the underlying malignancy itself [27]. These associations suggest that chronic inflammation, immune dysregulation, or paraneoplastic phenomena may contribute to the development of the veno-occlusive process.

## 5. Clinical Presentation

Patients with PVOD typically present with nonspecific symptoms, including progressive exertional dyspnea, fatigue, and exercise intolerance, which are clinically indistinguishable from IPAH. As the disease advances, additional symptoms may develop, such as chest discomfort, right upper quadrant pain from hepatic congestion, cyanosis, and exertional syncope. Less common but clinically significant presentations include diffuse alveolar hemorrhage and sudden cardiac death. The presence of hypoxemia out of proportion to hemodynamic findings or acute pulmonary edema following vasodilator therapy should raise suspicion for PVOD over PAH.

The physical examination is often nonspecific and reflects signs of pulmonary hypertension and right heart strain. Findings may include a right ventricular heave, a loud P2, and a systolic murmur of tricuspid regurgitation. In advanced stages, patients may exhibit signs of overt right heart failure, including peripheral edema, hepatomegaly, and ascites. Bibasilar crackles may be present due to pulmonary edema, and digital clubbing, while uncommon in PAH, may be seen in PVOD, particularly in younger patients.

## 6. Diagnostic Approach

The diagnosis of PVOD remains challenging due to its clinical and hemodynamic overlap with PAH. Definitive diagnosis requires histopathologic confirmation, but the risks associated with surgical lung biopsy in patients with pulmonary hypertension often preclude tissue diagnosis. As such, a multi-modality approach incorporating clinical, radiographic, physiologic, and genetic findings is essential to establishing a presumptive diagnosis.

The diagnostic evaluation begins with a thorough clinical assessment. Key features that should raise suspicion for PVOD include severe resting hypoxemia often out of proportion to symptoms or hemodynamics, a positive family history of pulmonary hypertension (particularly in younger patients), a markedly reduced DLCO on pulmonary function testing, HRCT findings consistent with venous and capillary involvement, and development of acute pulmonary edema in response to vasodilator therapy. Laboratory studies, including autoimmune serologies and HIV testing, may help exclude secondary causes or associated connective tissue disease.

### 6.1. Echocardiography

Echocardiography is typically the initial screening tool for pulmonary hypertension (PH), estimating pulmonary artery pressures and assessing right ventricular function [28]. While it can suggest the presence of PH, it cannot differentiate PVOD from other forms of PH.

### 6.2. High-Resolution Computed Tomography (HRCT)

**Figure 1:**
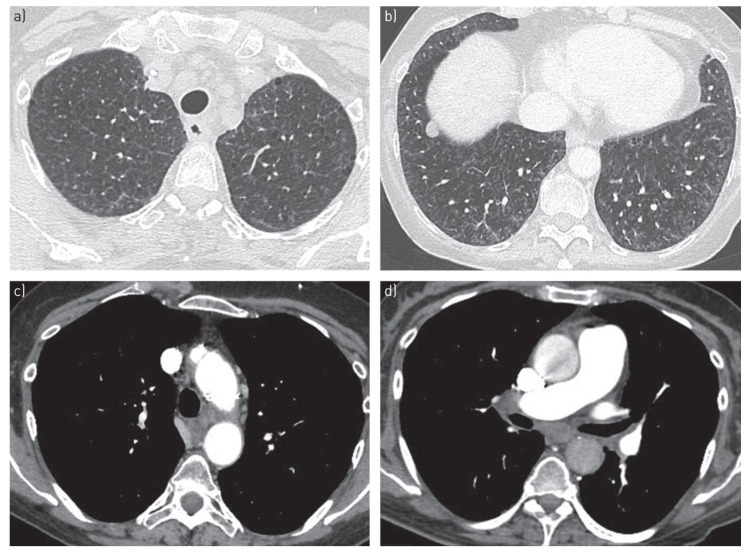
HRCT of patient with PVOD. Images (**a**,**b**) display centrilobular ground-glass opacities. Images (**c**,**d**) display mediastinal lymphadenopathy.

Imaging, particularly high-resolution computed tomography (HRCT) of the chest, is a key component of the noninvasive evaluation of suspected PVOD. Although not diagnostic in isolation, HRCT can reveal characteristic radiographic features that raise clinical suspicion. A classic triad—comprising centrilobular ground-glass opacities, smooth interlobular septal thickening, and mediastinal lymphadenopathy—is present in the majority of patients, with most exhibiting at least two of these findings Figure 1 [8,29,30,31].

These imaging features reflect the venular and capillary involvement that distinguishes PVOD from PAH, which typically lacks septal thickening and lymph node enlargement.

Additional HRCT findings may include small bilateral pleural effusions, mosaic attenuation or air-trapping in advanced stages, and enlargement of the pulmonary arteries due to coexisting pulmonary hypertension. While HRCT plays an essential role in the diagnostic workup, it cannot definitively establish the diagnosis. However, in the appropriate clinical context, HRCT findings consistent with PVOD may support a presumptive diagnosis, especially when lung biopsy is contraindicated.

### 6.3. Ventilation/Perfusion (V/Q) Scan

Ventilation/perfusion (V/Q) scanning has historically been thought to demonstrate unmatched perfusion defects in PVOD, mimicking chronic thromboembolic pulmonary hypertension. However, more recent data indicate that the vast majority of PVOD patients have normal V/Q scans [32]. As such, V/Q scan is not considered reliable for distinguishing PVOD from PAH and should not be used as a discriminating diagnostic tool in this context [24].

### 6.4. Pulmonary Function Testing

Pulmonary function testing provides important noninvasive clues in suspected PVOD. The hallmark finding is a markedly reduced diffusing capacity for carbon monoxide (DLCO), often below 40% predicted, which is substantially lower than in idiopathic pulmonary arterial hypertension (IPAH) or scleroderma-associated PAH. Across multiple studies, PVOD patients demonstrate mean DLCO values near 35% of predicted, compared with 45–60% in IPAH and similar ranges in scleroderma-associated PAH [30,31,33,34,35].

In IPAH, a DLCO < 45% is unusual and should prompt consideration of PVOD or concurrent parenchymal lung disease [33,36]. Although DLCO alone cannot distinguish PVOD from other PAH subtypes, a disproportionately low DLCO with preserved lung volumes strongly supports the diagnosis and should trigger further evaluation with high-resolution imaging and genetic testing.

### 6.5. Hemodynamic Profile

Although PVOD involves extensive obstruction of the post-capillary pulmonary venous system, both heritable and non-heritable forms typically present with a pre-capillary hemodynamic profile on right heart catheterization (RHC). Characteristic findings include a mPAP ≥ 20 mmHg, PCWP of ≤15 mmHg, and a PVR > 2 Wood units. These parameters render PVOD indistinguishable from PAH by hemodynamics alone [31].

This apparent paradox is due to the mechanics of PCWP measurement. During balloon occlusion, flow is halted proximal to the catheter tip, creating a static column of blood that reflects pressure in proximal pulmonary veins of relatively large caliber. In PVOD, however, it is the small venules and septal veins that are predominantly affected. As a result, the measured PCWP does not accurately reflect the elevated pressures generated by widespread obstruction in the distal pulmonary venous network. Left atrial pressure often remains normal, further obscuring the presence of post-capillary pathology. In some cases, PCWP may fall within a borderline range (e.g., 12–15 mmHg), but values rarely exceed the threshold for post-capillary hypertension unless there is coexisting left heart disease. When feasible, obtaining PCWP measurements in multiple pulmonary artery branches may improve accuracy [6].

Acute vasoreactivity testing is contraindicated in PVOD. Administration of agents such as inhaled nitric oxide or intravenous prostacyclin can lead to rapid-onset pulmonary edema, a potentially fatal complication. This occurs because vasodilation of pulmonary arterioles increases blood flow into a pulmonary vascular bed with fixed outflow obstruction, resulting in elevated transcapillary hydrostatic pressure and fluid transudation into the alveolar and interstitial spaces [6,37]. This mechanism stands in stark contrast to PAH, where vasoreactivity testing can identify a subset of patients who benefit from high-dose calcium channel blockers. Although not pathognomonic, the development of pulmonary edema following vasodilator administration is highly suggestive of PVOD.

### 6.6. Genetic Testing

In patients with suspected PVOD, particularly those with early-onset disease, a positive family history, or suggestive radiographic findings, genetic testing for biallelic mutations in *EIF2AK4* is recommended. Identification of pathogenic *EIF2AK4* variants is considered diagnostic of heritable PVOD, eliminating the need for histologic confirmation in most cases. Genetic counseling should be offered to affected individuals and at-risk family members to guide screening and reproductive planning. This can be achieved through next-generation sequencing panels that are often available in specialized pulmonary hypertension centers.

Biallelic *EIF2AK4* mutations are detected in nearly all familial PVOD cases and in approximately 25–31% of sporadic cases, depending on diagnostic criteria and cohort composition. In one study, *EIF2AK4* mutations were identified in all 13 families with heritable PVOD and in 25% of histologically confirmed sporadic cases [10,38,39]. These findings establish *EIF2AK4* as the major gene linked to PVOD and underscore its value as a critical diagnostic biomarker in both familial and sporadic forms. Detection rates may vary depending on whether the diagnosis is based on clinical suspicion, histopathology, or molecular testing.

While *EIF2AK4* remains the only gene with a well-established, causative role in PVOD, testing for additional genes may be considered in select cases, particularly those with atypical features or unclear family history. Rare variants in PAH-associated genes such as *BMPR2*, *TBX4*, and *SMAD9* have been reported in patients with PVOD-like phenotypes, although their clinical significance in this context remains uncertain [13,14,15,16,17,18]. These mutations are more commonly linked to heritable PAH, and their presence in PVOD likely reflects phenotypic overlap or modifier effects rather than direct causation.

Although VEGF pathway dysregulation has been implicated in the pathobiology of PVOD, particularly in capillary proliferation and endothelial remodeling, there is currently no strong evidence supporting a role for germline mutations in VEGF-related genes in the development of PVOD.

In summary, *EIF2AK4* is the sole gene currently considered diagnostic of heritable PVOD. Other genetic variants may contribute to phenotypic heterogeneity, and their inclusion in initial genetic workup may aid in distinguishing PVOD from PAH in select cases.

### 6.7. Role of Bronchoscopy and BAL

Bronchoscopy with bronchoalveolar lavage (BAL) may provide supportive evidence in the evaluation of suspected PVOD, particularly through the detection of occult alveolar hemorrhage. This is typically demonstrated by the presence of hemosiderin-laden macrophages in the BAL fluid, reflecting capillary and venous involvement that is characteristic of PVOD but uncommonly seen in idiopathic PAH [33].

However, while suggestive, this finding is not specific to PVOD and may also be observed in other conditions involving alveolar hemorrhage. As such, BAL is not required for diagnosis and should be interpreted in conjunction with other clinical, radiographic, and hemodynamic data.

Transbronchial biopsy is not recommended due to the high risk of procedural complications and limited diagnostic value [33].

### 6.8. Surgical Lung Biopsy

Surgical lung biopsy is the histopathologic gold standard for diagnosing PVOD, revealing venular obliteration with intimal fibrosis and secondary capillary proliferation. In practice, however, biopsy is strongly discouraged in patients with pulmonary hypertension as pulmonary vascular manipulation in this patient population can trigger fulminant pulmonary edema or acute right-heart failure, leading to markedly elevated peri-operative mortality [8,30,34,40,41].

Consequently, tissue sampling is reserved for exceptional circumstances in which non-invasive studies are inconclusive yet a definitive diagnosis would alter management, such as atypical radiographic patterns, suspicion of alternative interstitial, vasculitic, or neoplastic processes, or other discordant clinical features. When biopsy is unavoidable, video-assisted thoracoscopic surgery (VATS) is preferred owing to its lower morbidity and shorter recovery; even so, it should proceed only after multidisciplinary discussion and meticulous peri-operative planning.

**Figure 2:**
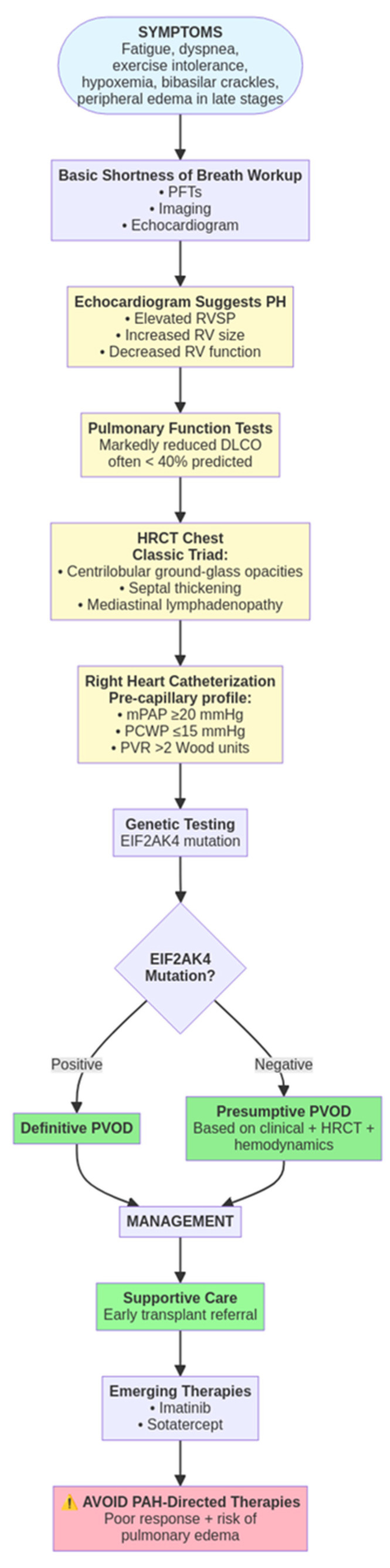
Diagnostic algorithm of PVOD.

When histologic confirmation is required for research, epidemiologic, or definitive diagnostic purposes, post-mortem lung examination provides unequivocal evidence of PVOD and may be particularly useful for familial risk assessment and genetic counseling [30,42] (Figure 2).

## 7. Differential Diagnosis

Given its clinical, radiographic, and hemodynamic overlap with other forms of pulmonary hypertension and diffuse lung disease, PVOD presents a significant diagnostic challenge. The differential diagnosis primarily includes IPAH, but also encompasses conditions associated with interstitial lung disease, pulmonary capillary hemangiomatosis (PCH), and chronic thromboembolic pulmonary hypertension (CTEPH). Accurate differentiation is critical, as the use of pulmonary vasodilators in PVOD can lead to life-threatening pulmonary edema.

### 7.1. Idiopathic Pulmonary Arterial Hypertension (IPAH)

Idiopathic PAH remains the most common diagnostic consideration in patients with suspected PVOD, owing to their shared clinical presentation, age distribution, and pre-capillary hemodynamic profile. However, several distinguishing features favor PVOD and should prompt consideration of this diagnosis.

Key differentiating findings include HRCT abnormalities such as smooth interlobular septal thickening, centrilobular ground-glass opacities, and mediastinal lymphadenopathy—features that are uncommon in IPAH. Most notably, the development or worsening of pulmonary edema following initiation of PAH-targeted therapy is a hallmark of PVOD and should raise immediate concern. Unlike PVOD, patients with IPAH may demonstrate positive vasoreactivity and typically lack imaging evidence of pulmonary venous or lymphatic congestion. Table 1.

### 7.2. Pulmonary Capillary Hemangiomatosis (PCH)

PCH is a rare vascular disorder characterized by uncontrolled proliferation of pulmonary capillaries. It is considered by many to lie on a histologic and clinical spectrum with PVOD, more recently recognized more-so as an overlapping entity, particularly in cases with *EIF2AK4* mutations [43,44].

Radiographically, PCH and PVOD demonstrate similar findings on HRCT, including ground-glass opacities and septal thickening. However, PCH may present with more diffuse or nodular ground-glass opacities, reflecting its predominant capillary involvement [44]. This histologic distinction between the two entities lies in the primary site of vascular remodeling (capillaries in PCH versus venules in PVOD). Despite this, the clinical relevance of this distinction is limited, as both conditions share similar presentations, genetic profiles, prognostic implications, and management strategies [43,44].

### 7.3. Interstitial Lung Disease (ILD)

ILDs associated with pulmonary hypertension, such as those seen in systemic sclerosis, hypersensitivity pneumonitis, and connective tissue disease-associated ILD, can closely resemble PVOD, particularly in terms of clinical presentation. Both entities may manifest with severe resting hypoxemia and a markedly reduced DLCO, reflecting impaired gas exchange from microvascular and/or interstitial involvement.

Despite these similarities, important distinctions exist. Restrictive ventilatory defects and reduced lung volumes are more characteristic of ILD, whereas these findings are generally absent or mild in PVOD. On HRCT, both conditions may demonstrate ground-glass opacities and interlobular septal thickening, but ILD-associated pulmonary hypertension more commonly features reticulation, traction bronchiectasis, and honeycombing, indicative of underlying parenchymal fibrosis, whereas PVOD typically demonstrates the classic triad of imaging findings mentioned earlier.

Finally, PVOD (and PCH) is uniquely associated with biallelic *EIF2AK4* mutations, a genetic hallmark not observed in ILD-associated pulmonary hypertension [10].

### 7.4. Chronic Thromboembolic Pulmonary Hypertension (CTEPH)

CTEPH is another form of pre-capillary PH that can mimic PVOD. Both can present with progressive dyspnea and right heart failure. However, CTEPH is characterized by chronic obstruction of the pulmonary arteries by organized thrombi [45]. V/Q scanning is the primary screening tool for CTEPH, typically showing segmental or subsegmental perfusion defects, which are usually absent or non-specific in PVOD [46]. Pulmonary angiography and right heart catheterization with selective pulmonary angiography are confirmatory for CTEPH [46]. Interestingly, both conditions can coexist, as evidenced by one case report confirming both CTEPH and PVOD on surgical pathology following successful double lung transplant [47]. This highlights the complexity of differential diagnosis and the potential for overlapping pathologies.

## 8. Management Strategies

The management of PVOD remains challenging due to its limited therapeutic options, risk of harm from conventional PAH-directed therapies, and poor prognosis. Unlike IPAH, where targeted vasodilator therapies form the cornerstone of treatment, PVOD requires a more nuanced and cautious approach. The current management paradigm centers on supportive care, early referral for lung transplantation, and avoidance of therapies that may provoke pulmonary edema [4,6,40].

### 8.1. Supportive Measures

Initial management should prioritize oxygenation, volume optimization, and symptom control. Most patients require long-term supplemental oxygen due to significant resting hypoxemia; maintaining adequate oxygen saturation is essential to reduce hypoxic pulmonary vasoconstriction and mitigate right ventricular strain. Diuretics may be used to manage right heart failure and prevent fluid overload, but should be administered cautiously to avoid excessive preload reduction, which may precipitate hypotension or compromise cardiac output.

Physical activity should be individualized based on functional status, and patients should be closely monitored for signs of clinical deterioration, including progressive dyspnea, worsening hypoxemia, or evidence of right ventricular dysfunction.

Anticoagulation is generally not recommended, given the absence of a thrombotic component in PVOD and the potential risk of bleeding in the setting of occult alveolar hemorrhage. Exceptions may include rare cases in which PVOD coexists with objectively confirmed pulmonary embolism, warranting individualized risk-benefit assessment [46,48].

### 8.2. PAH-Targeted Therapy-Use with Extreme Caution

Unlike in PAH, vasodilator therapy in PVOD carries a high risk of inducing life-threatening pulmonary edema, due to increased blood flow into an already obstructed venous system. Agents such as prostacyclins, endothelin receptor antagonists, and phosphodiesterase-5 inhibitors have been associated with acute decompensation in PVOD patients. If used at all, these agents should be initiated at low doses, under close inpatient monitoring, and only in select cases where there is no immediate access to transplantation and symptoms are rapidly progressing.

In select cases, PAH-targeted therapy may be used cautiously as a bridge to lung transplantation. The intent of such therapy is limited to short-term symptomatic stabilization rather than disease modification. This is typically performed under close inpatient monitoring and should be initiated only in specialized pulmonary hypertension centers managed by experienced multidisciplinary teams.

Inhaled therapies (e.g., inhaled nitric oxide or inhaled prostacyclins) may be considered in certain patients with severe symptoms, as their localized pulmonary vasodilation may have a more favorable safety profile. However, data supporting their use are limited, and all PAH-directed therapies should be approached with caution.

Should PAH-targeted therapy be initiated in patients with PVOD, pulmonary edema may develop within hours to days, manifesting as worsening dyspnea, new or progressive crackles on auscultation, increased oxygen requirements, and new bilateral alveolar infiltrates on chest imaging. Management is primarily supportive, including supplemental oxygen and diuresis as indicated, but the initial and most critical step is recognition with cautious reduction or discontinuation of the precipitating PAH-targeted agent.

### 8.3. Lung Transplantation

Lung transplantation remains the only definitive treatment for PVOD and should be considered early in the disease course, ideally at the time of diagnosis. Due to the condition’s rapid progression and limited responsiveness to medical therapy, timely referral to a transplant center is critical. Both single and bilateral lung transplantation have been successfully performed, though bilateral transplantation is generally preferred, as it replaces all affected pulmonary vasculature and reduces the risk of persistent or recurrent pulmonary hypertension in the native lung [49,50].

Single lung transplantation may be considered in select scenarios, such as pediatric patients or when donor organ availability is limited. However, this approach carries increased risks, including persistent disease in the native lung, ventilation-perfusion mismatch, and pulmonary edema due to ongoing post-capillary obstruction.

Waitlist mortality is high, underscoring the importance of close monitoring while awaiting transplantation [51,52]. Preoperative optimization and careful perioperative management are essential, especially in patients with severe hypoxemia or right heart dysfunction. Despite the severity of PVOD, post-transplant outcomes are generally favorable, with contemporary studies reporting 1-year survival rates of 81–95% and 3- to 5-year survival rates of 58–84%, comparable to those seen in patients transplanted for IPAH [49,50,51]. Interestingly, PVOD patients often experience shorter waitlist times compared to PAH, likely reflecting more severe baseline disease. While postoperative ECMO support is required more frequently in PVOD, long-term survival outcomes are comparable to those of PAH patients [53].

Combined heart-lung transplantation is reserved for patients with severe, irreversible cardiac involvement that would preclude recovery following isolated lung transplantation [54,55]. Meta-analysis comparing double-lung transplantation and combined heart-lung transplantation have shown no significant differences in 1-, 3-, 5-, or 10-year survival rates. Furthermore, no difference was noted in rates of chronic lung allograft dysfunction or perioperative complications [56]. Registry data does confirm higher early post-transplant mortality for patients who undergo combined heart-lung transplant versus double lung transplant, but long-term survival rates are similar [55,57]. Other post-transplant complications such as graft dysfunction, infection, and chronic allograft dysfunction occur at similar rates between lung and combined heart-lung transplantation, though combined heart-lung transplantation may have a lower incidence of bronchiolitis obliterans syndrome and cardiac allograft vasculopathy [57].

In summary, lung transplantation alone is preferred for PVOD unless there is significant cardiac disease, with similar survival and complication rates to combined heart-lung transplantation.

### 8.4. Investigational and Emerging Therapies

Although there are no approved disease-specific medical treatments for PVOD, a small number of emerging therapies have shown potential in early-stage studies. One investigational target is the *EIF2AK4* pathway, which plays a central role in heritable PVOD pathogenesis. Preclinical models are exploring compounds that modulate the integrated stress response pathway, aiming to restore endothelial homeostasis and prevent venular remodeling. While still in experimental stages, this represents a rational, genetically informed approach to disease modification.

Imatininb, a tyrosine kinase inhibitor with antiproliferative effects, has been studied in pulmonary vascular diseases due to its ability to inhibit platelet-derived growth factor (PDGF) signaling. Limited case reports have described its off-label use in PVOD, with mixed clinical responses [58,59,60]. Its use remains investigational and must be approached cautiously, given the risk of exacerbating pulmonary edema in this population.

Sotatercept, a novel fusion protein targeting the transforming growth factor-β (TGF-β) superfamily, has shown efficacy in pulmonary arterial hypertension and although PVOD is pathophysiologically distinct from IPAH, shared features of vascular remodeling raise the possibility that sotatercept or related agents may have therapeutic value in PVOD. However, currently only a single case report exists detailing its use in PVOD [61]. As with other PAH-directed therapies, the risk of pulmonary edema also exists.

Comparative safety data between Imatinib and Sotatercept remain limited, but both agents carry a persistent risk of pulmonary edema. Their use should therefore be approached with caution and reserved for individualized application, ideally within research protocols or under close clinical supervision until more definitive outcome data become available.

Further research is essential to determine whether these or other pathway-specific therapies can be safely and effectively integrated into the management of PVOD.

## 9. Prognosis

PVOD is associated with a significantly worse prognosis compared to other forms of pulmonary hypertension, including IPAH. In the absence of definitive treatment, the disease follows a relentlessly progressive course, often culminating in right heart failure and death. Several factors contribute to this poor prognosis, including diagnostic delay, the lack of effective medical therapy, and the potential for rapid decompensation. Reported median survival from diagnosis is typically less than two years, markedly shorter than that observed in IPAH [7,62]. In contemporary PAH registries, 1-year survival for IPAH is approximately 86–90%, 2-year survival is about 80–84%, and 5-year survival is 61–65% [63,64,65,66,67]. In contrast, PVOD patients have markedly worse outcomes, with 1-year survival rates reported as low as 50–60%, and 5-year survival often less than 20–30% [7,31,68].

As stated earlier, lung transplantation remains the only curative intervention. However, rapid clinical deterioration frequently precludes timely transplant, contributing to elevated waitlist mortality [51,52]. Thus, early transplant referral, close monitoring, and proactive transplant evaluation, with eventual optimal perioperative management remain critical to achieving successful outcomes.

## 10. Future Directions

The landscape of PVOD research is rapidly evolving, driven by advances in genetics, molecular biology, and pulmonary vascular imaging. However, the disease remains in an area of significant unmet need. Several key domains will shape future progress in the diagnosis, treatment, and understanding of PVOD.

Improved diagnostic strategies are urgently needed. Current approaches rely heavily on imaging and clinical suspicion, with presumptive diagnosis often derived following poor response to empiric PAH therapy and definitive diagnosis limited by the risks of lung biopsy. Future efforts should focus on developing noninvasive biomarkers—including blood-based assays, imaging-derived features, and artificial intelligence-driven diagnostic models—to distinguish PVOD from PAH early in the disease course and avoid the dangers of inappropriate therapy.

Targeted therapeutic development remains a top priority. Agents that modulate the *EIF2AK4*-integrated stress response pathway, address abnormal endothelial proliferation, or reverse venular fibrosis are under investigation. Drugs such as imatinib and sotatercept, though requiring careful risk-benefit assessment, may offer benefit in select cases. As preclinical tools improve, therapeutic screening in PVOD-specific models will be increasingly feasible.

Genetic research will continue to shape the field. While *EIF2AK4* mutations are well established in heritable PVOD, further study on non-*EIF2AK4* variants may uncover additional genetic drivers or modifiers of disease. Understanding genotype-phenotype correlations could enhance both diagnostic and prognostication and support the development of personalized treatment strategies.

Progress in PVOD will also depend on collaborative infrastructure. The continued establishment of international patient registries and biobanks will facilitate larger-scale studies, support rare disease clinical trials, and provide critical biospecimens for translational research. Given the small patient population, muti-center collaboration will be essential to generate robust clinical data.

Finally, increasing awareness among clinicians is crucial. Educational efforts should emphasize the distinct clinical and radiographic features of PVOD, the risks of empiric PAH therapy, and the importance of early referral to specialized pulmonary hypertension centers capable of lung transplantation.

Together, these future directions represent a multi-pronged approach that integrates innovation in diagnostics, therapeutics, and systems-level coordination. Sustained research investment and cross-disciplinary collaboration will also be essential in altering the natural history of PVOD.

## 11. Conclusions

Pulmonary veno-occlusive disease is a rare yet devastating pulmonary vascular disorder that demands heightened clinical vigilance, nuanced diagnostic evaluation, and timely referral for lung transplantation. While supportive care and transplantation remain the cornerstone of management, our evolving understanding of the disease’s genetic and pathophysiologic basis may begin to guide research efforts aimed at identifying future diagnostic and therapeutic strategies. Continued multidisciplinary collaboration across clinical research, translational science, and international registry development will be essential to render these insights into earlier diagnosis, effective interventions, and ultimately, improved outcomes for patients with PVOD.

## Figures and Tables

**Table 1:** Key distinctions between pulmonary veno-occlusive disease (PVOD) and idiopathic pulmonary arterial hypertension (IPAH).

Feature	Pulmonary Veno-Occlusive Disease (PVOD)	Idiopathic Pulmonary Arterial Hypertension (IPAH)
Primary site of pathology	Post-capillary venules and small pulmonary veins; intimal fibrosis and occlusion	Pre-capillary arterioles; medial hypertrophy and plexiform lesions
Genetic associations	Biallelic EIF2AK4 mutations; rare overlap with BMPR2, TBX4, SMAD9 variants	BMPR2, TBX4, ACVRL1, SMAD9, CAV1, ENG, others
Histopathology	Venular fibrosis and occlusion; capillary congestion; absence of plexiform lesions	Arteriolar remodeling with plexiform lesions and medial hypertrophy
Hemodynamics (RHC)	Pre-capillary PH profile (mPAP ≥ 20 mmHg, PCWP ≤ 15 mmHg, PVR > 2 WU), indistinguishable from IPAH	Pre-capillary PH profile (mPAP ≥ 20 mmHg, PCWP ≤ 15 mmHg, PVR > 2 WU)
HRCT findings	Classic triad: centrilobular ground-glass opacities, smooth interlobular septal thickening, mediastinal lymphadenopathy	Enlarged pulmonary arteries; otherwise often normal parenchyma
Response to PAH therapies	Poor; vasodilators may precipitate pulmonary edema	Often improves with PAH-targeted therapy; vasoreactivity testing may identify responders
Bronchoalveolar lavage	Hemosiderin-laden macrophages (evidence of occult hemorrhage reflecting capillary leak and venous involvement)	Typically absent
V/Q scan	Usually normal	Usually normal
Prognosis	Rapidly progressive; median survival < 2 years without transplant	Variable; median survival 5–7 years with therapy
Definitive therapy	Lung transplantation (curative)	Mainstay is medical therapy; transplant reserved for refractory cases

Comparison of pulmonary veno-occlusive disease (PVOD) and idiopathic pulmonary arterial hypertension (IPAH). Abbreviations: HRCT = high-resolution computed tomography; mPAP = mean pulmonary artery pressure; PCWP = pulmonary capillary wedge pressure; PVR = pulmonary vascular resistance; V/Q = ventilation/perfusion; WU = Wood units.

## Data Availability

No new data were created or analyzed in this study.
